# Progression of Paravalvular Leak and Hemolytic Anemia After Implantation of Fifth-Generation Balloon-Expandable Transcatheter Heart Valve

**DOI:** 10.1016/j.jaccas.2025.103410

**Published:** 2025-03-26

**Authors:** Nozomu Kanehama, Ryo Ninomiya, Kengo Tosaka, Yoshifumi Nakajima, Tetsuya Fusazaki, Yoshihiro Morino

**Affiliations:** Division of Cardiology, Department of Internal Medicine, Iwate Medical University, Yahaba, Iwate, Japan

**Keywords:** aortic valve, occluder, valve replacement

## Abstract

**Background:**

Progression of paravalvular leak (PVL) and hemolytic anemia are rare complications of newer-generation transcatheter heart valves used for transcatheter aortic valve replacement.

**Case Summary:**

An 81-year-old man presented with worsening shortness of breath 1 month after implantation of SAPIEN 3 Ultra RESILIA (S3UR). Echocardiography revealed PVL progression and blood tests indicated hemolytic anemia. Percutaneous plug closure was successfully performed, reducing the PVL. His symptoms and hemolytic anemia improved.

**Discussion:**

The progression of PVL and hemolytic anemia in patients with S3UR has not been previously reported, and their mechanisms require further investigation. Percutaneous plug closure is a good treatment option for PVL after transcatheter aortic valve replacement.

**Take-Home Message:**

This case demonstrates that PVL progression and hemolytic anemia remain a potential risk after S3UR implantation, which can effectively be treated through percutaneous plug closure.

## Visual Summary

**Figure:**
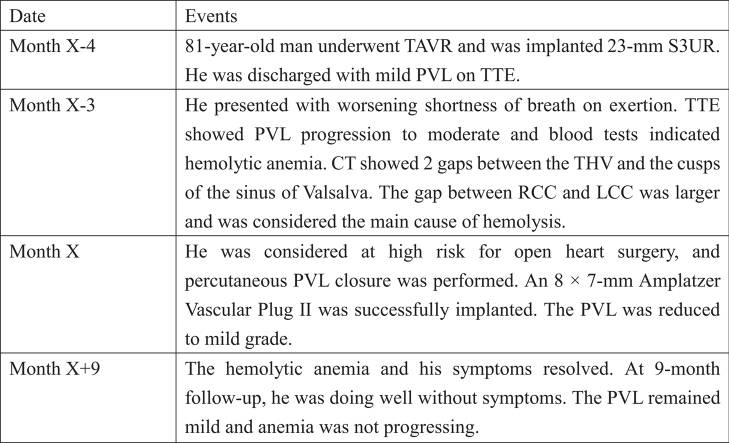

## History of Presentation

An 81-year-old man visited the outpatient clinic complaining of worsening shortness of breath on exertion 1 month after undergoing transcatheter aortic valve replacement (TAVR). The patient’s blood pressure was 132/62 mm Hg, and his heart rate was 85 beats/min with a regularly irregular rhythm. A diastolic murmur (Levine grade 2/6) was detected in the third left sternal border.

**Take-Home Messages**

• This case demonstrates the potential risk of PVL progression and hemolytic anemia following S3UR implantation, which has not been previously reported.
• Percutaneous plug closure is a suitable treatment option for these complications.

## Past Medical History

**Figure 1:**
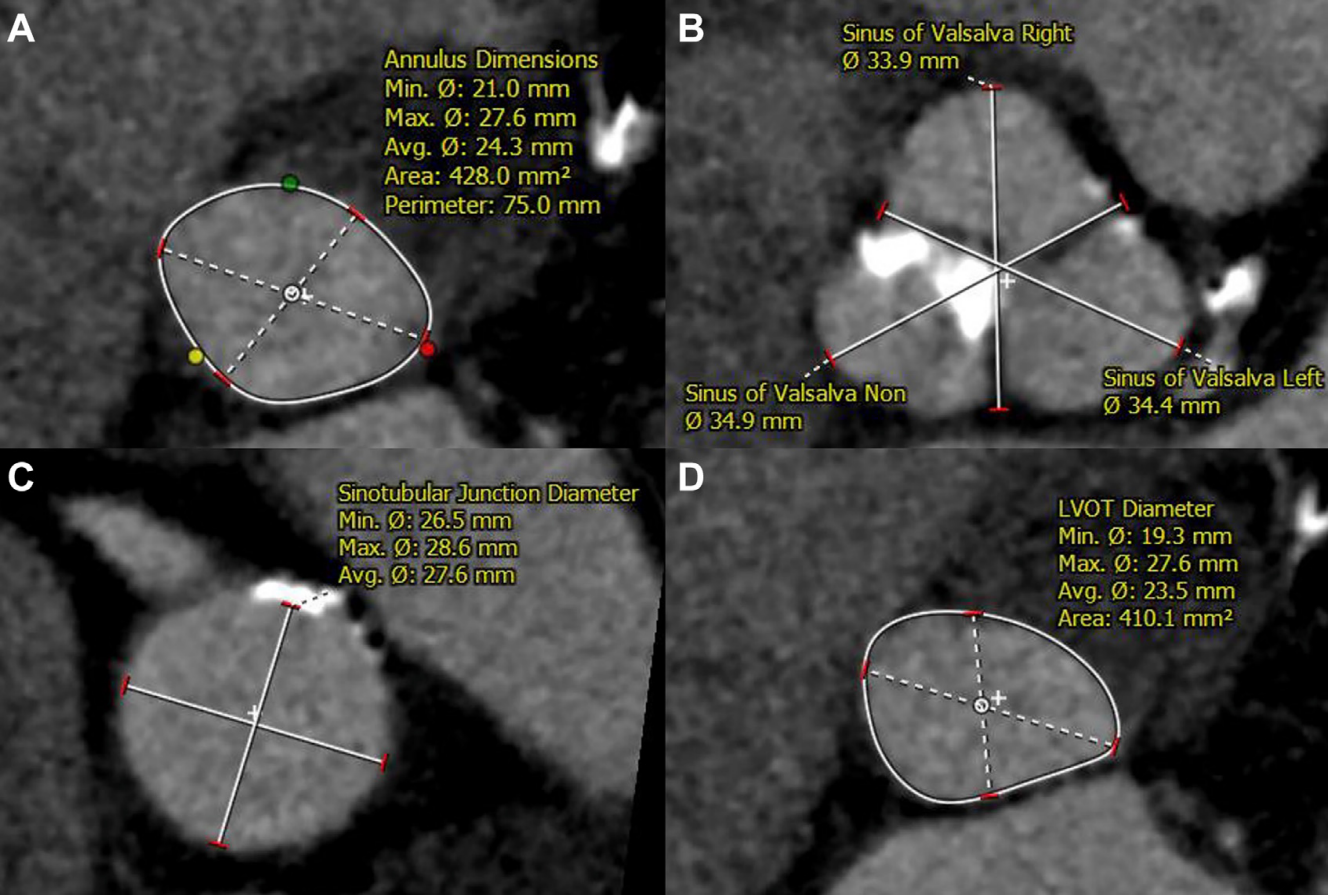
Computed Tomography Measurements of the Aortic Valve Complex Before Transcatheter Aortic Valve Replacement (A) Annulus. (B) Sinus of Valsalva. (C) Sinotubular junction. (D) Left ventricular outflow tract.

**Figure 2:**
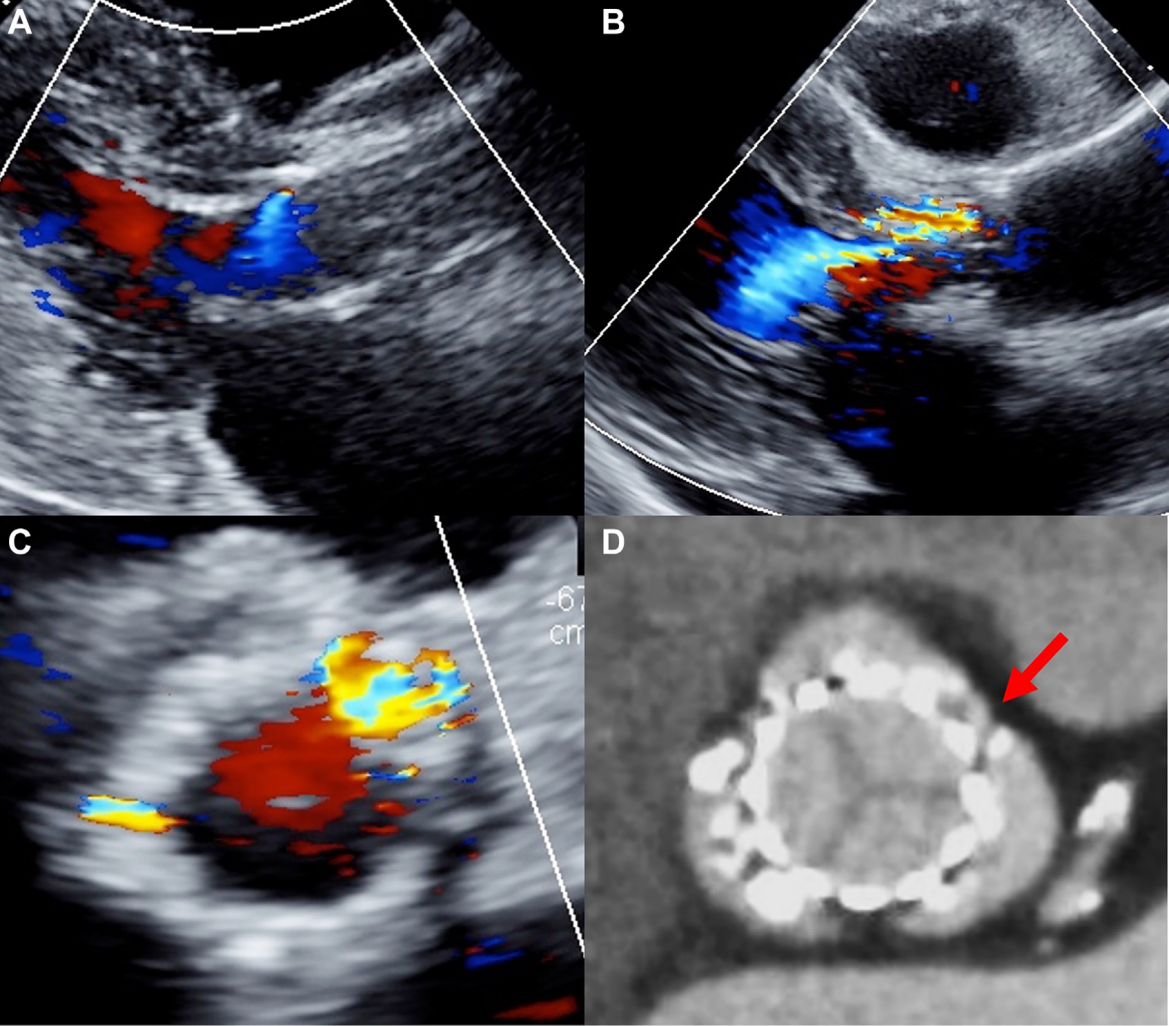
Progression of Pravalvular Leak (A) Transthoracic echocardiography at discharge. Paravalvular leak was mild. (B and C) Transthoracic echocardiography at 1 month (B: long-axis view, C: short-axis view). Paravalvular leak progressed to moderate. Main paravalvular leak originated from 1 o’clock position between left and right coronary cusps. (D) Computed tomography showed the gap between left and right coronary cusps (red arrow).

The patient had severe aortic stenosis with shortness of breath and was scheduled to undergo TAVR because of older age and mild frailty. His medical history included the presence of hypertension. Preoperative evaluation revealed 2-vessel coronary artery disease and severe stenosis of the right internal carotid artery. Computed tomography (CT) imaging is shown in Video 1 and measurements of the aortic valve complex are shown in Figure 1. The annulus area was 428 mm^2^. Although this was borderline between 23 and 26 mm for SAPIEN 3 Ultra RESILIA (S3UR), a smaller 23-mm valve was selected to avoid the risk of the annular rupture. TAVR was performed through the transfemoral approach, and a 23-mm S3UR was implanted at a nominal volume. Post-dilatation was added with +1 mL because of a residual paravalvular leak (PVL). The PVL was reduced to mild grade (Videos 2 and 3), and the procedure was completed without complications. The patient was discharged with mild PVL on transthoracic echocardiography (TTE) (Figure 2A).

## Differential Diagnosis

The following diagnoses were considered possible causes of shortness of breath: acute heart failure, arrhythmia, or prosthetic valve dysfunction (deterioration of valve leaflets, leaflet thrombosis, PVL).

## Investigations

**Figure 3:**
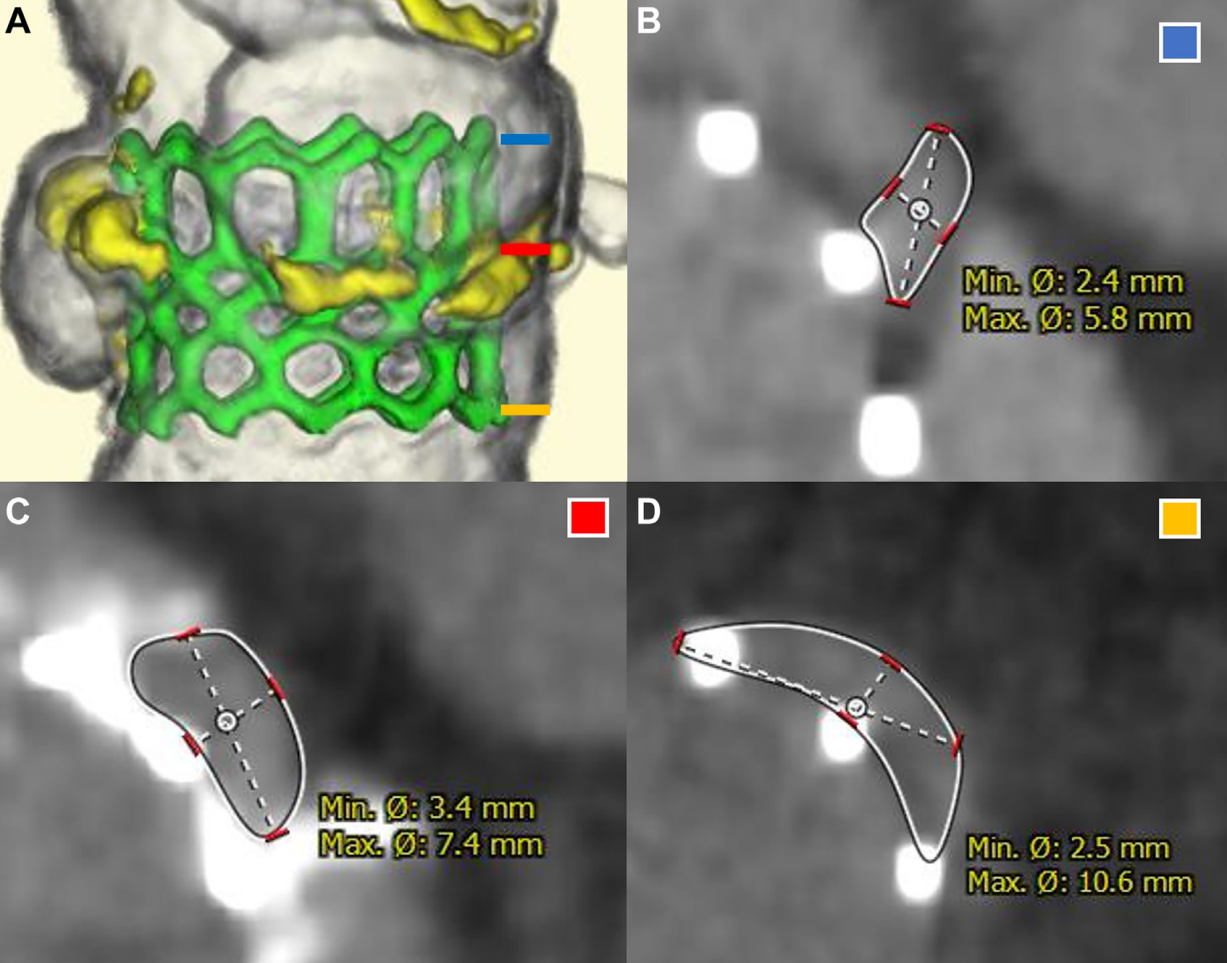
Three-Dimensional Anatomy of Gap (A) Three-dimensional positioning of native aortic valve and transcatheter heart valve. Stent frame of transcatheter heart valve is illustrated in green. (B) Aortic side (level of blue line). (C) Mid portion (level of red line). (D) Left ventricular side (level of yellow line).

Blood tests showed no change in the N-terminal prohormone of brain natriuretic peptide, but progression of anemia with an intravascular hemolytic pattern (elevated lactate dehydrogenase and reticulocytes, decreased haptoglobin, and presence of schistocytes) was evident. Electrocardiography revealed bigeminy with premature atrial contraction. TTE showed worsening PVL, which had progressed to moderate grade (Figures 2B and 2C). However, the valve leaflet motion was good, and there was no transvalvular regurgitation. Transesophageal echocardiography also showed exacerbated PVL compared with that of postoperative echocardiography (Video 4). CT showed no thrombus at the leaflets of the transcatheter heart valve (THV), but there were 2 gaps between the THV and the cusps of the sinus of Valsalva (SOV) (Figure 2D, Video 5). The gap between left and right coronary cusps was larger and was considered the main cause of hemolysis. The gap measured 2.4 × 5.8 mm (aortic side), 7.4 × 3.4 mm (mid portion), and 10.6 × 2.5 mm (left ventricular side) (Figure 3).

## Management

**Figure 4:**
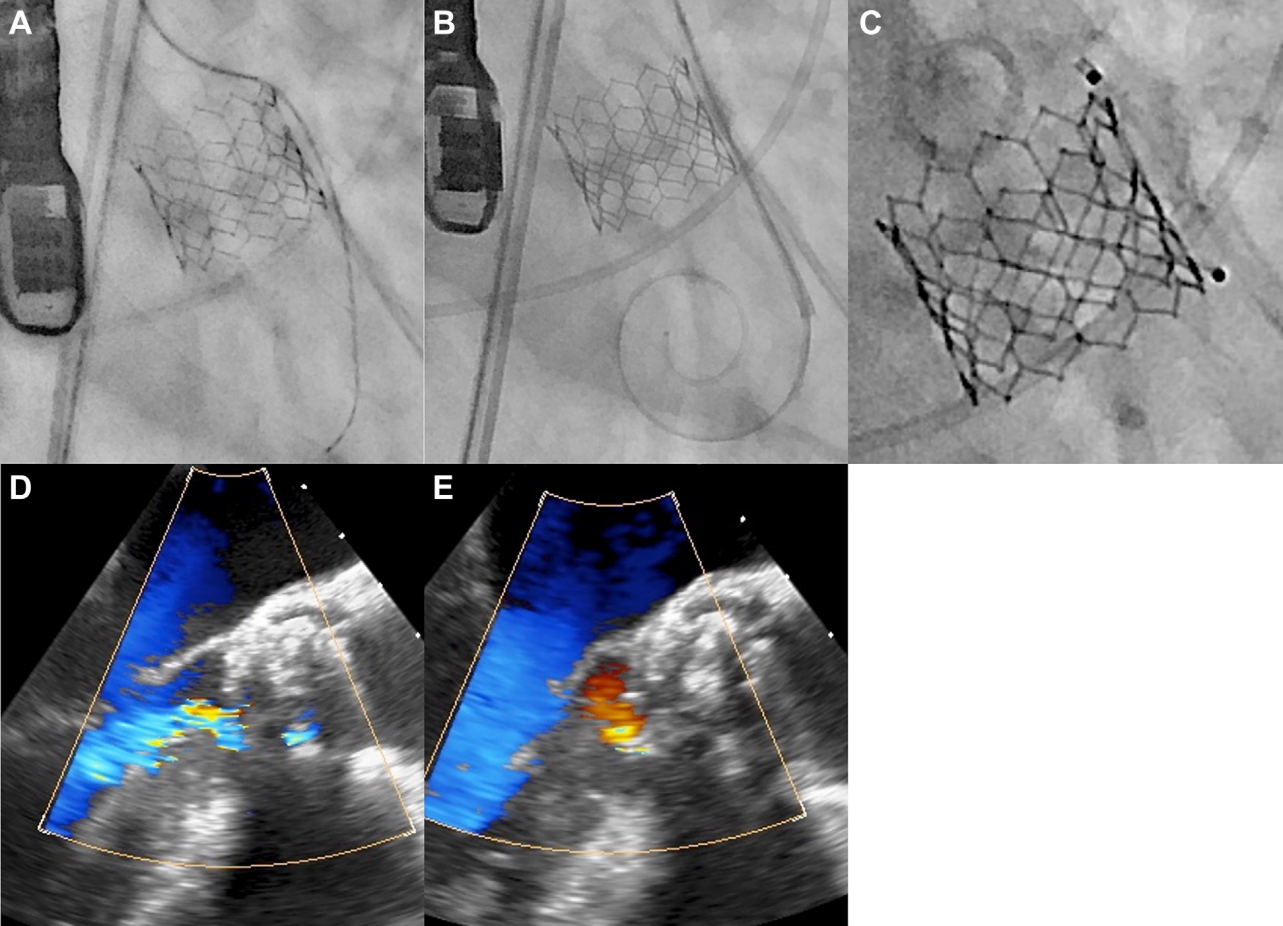
Procedure of Percutaneous Plug Closure (A) A 0.035-inch hydrophilic guidewire with 5-F guiding sheath and 4-F Amplatz-Left-2 catheter passed the gap. (B) Guidewire was exchanged to Safari XS and 6-F straight catheter passed the gap. (C) An 8 × 7-mm Amplatzer Vascular Plug II was implanted. (D) Transesophageal echocardiography of pre-procedure. (E) Transesophageal echocardiography after implantation of vascular plug.

The symptoms and hemolytic anemia of the patient did not improve with conservative treatment; therefore, an invasive intervention was decided. Given his advanced age, mild frailty, and severe stenosis of the right internal carotid artery, he was considered at high risk for open heart surgery, and percutaneous PVL closure was performed. The procedure was performed under general anesthesia, transesophageal echocardiography guidance, and a transfemoral approach. The gap between left and right coronary cusps was crossed with a 0.035-inch hydrophilic guidewire with a 5-F guiding sheath and a 4-F Amplatz-Left-2 catheter (Figure 4A). The guidewire was exchanged with the Safari XS (Boston). The 5-F guiding sheath did not pass through the gap and was replaced with a 6-F straight catheter (Figure 4B). This catheter was passed through the gap, and an 8 × 7-mm Amplatzer Vascular Plug II (Abbott Cardiovascular) was successfully implanted (Figure 4C). The PVL was reduced to mild grade (Figures 4D and 4E, Video 6).

## Outcome and Follow-Up

Following percutaneous PVL closure, PVL was reduced from moderate to mild grade, and the hemolytic anemia improved, with subsequent disappearance of symptoms.

## Discussion

Although PVL after TAVR has been associated with poor prognosis, it has been mitigated by recent improvements in the outer skirt of THV. In the SAPIEN 3 (S3), a previous model of the balloon-expandable valves, only 1.4% had moderate or severe PVL at 1 month.^1^ The outer skirt of the S3UR was further improved by changing the material to textured polyethylene terephthalate and increasing the height by 40% compared with that of the S3, which is expected to reduce the PVL. In this case, PVL was mild immediately after THV implantation and at discharge but progressed to moderate 1 month later, resulting in clinically significant hemolytic anemia. Such a clinical course is uncommon and the underlying mechanisms require further verification.

First, there was a possibility of THV recoil, but in this case, the stent frame diameter measured by fluoroscopic imaging did not change immediately after the TAVR or during PVL closure. However, because it was not compared with CT imaging, accurate evaluation of the recoil was not possible. Second, underexpansion of the THV might have occurred. In this case, it was expanded by +1 mL. Considering the presence of residual PVL and the sufficient space in the SOV, further expansion by +2 mL or +3 mL might have been feasible. Third, the selected THV was relatively undersized compared with the size of the aortic valve complex. Although the annulus area of this case was 428 mm^2^, which was borderline between a 23- and 26-mm S3UR, the mean SOV diameter was 34.4 mm and the mean sinotubular junction diameter was 27.6 mm. Considering the size of the SOV and sinotubular junction, a 26-mm THV might have been more appropriate. However, the annular oversizing was 21%, increasing the risk of annular rupture. Therefore, we selected a 23-mm THV for this case, but it was undersized compared with the SOV diameter, resulting in a gap and subsequent PVL progression with hemolytic anemia. Finally, there might be concerns regarding the S3UR itself, such as its new outer skirt. In fact, with the S3, we did not encounter similar issues in situations like this case. The new outer skirt was modified from the S3 to a textured polyethylene terephthalate resembling “towel fabric.” It may have unexpectedly and dynamically changed in the body, resulting in PVL progression. The change in material may have also caused unexpected shear stress of the PVL jet, which may have accelerated hemolysis. However, these are only speculative and further investigation is needed, such as to demonstrate the frequency of PVL progression and hemolysis compared with the S3 in even larger cohorts or analyzing PVL jets using other imaging modalities or in vitro experiments.

PVL after TAVR requires an invasive intervention when refractory heart failure or hemolysis occurs. Although the American College of Cardiology/American Heart Association guidelines recommend percutaneous repair of surgical valves for PVL as Class 2a,^2^ there are no established guidelines for PVL after TAVR. Although surgical intervention is generally considered, percutaneous treatment is an option for high-risk cases. Reported percutaneous treatment options for PVL after TAVR include redo TAVR, balloon valvuloplasty, and plug closure.^3^ In this case, the gap to be closed was clearly visible on the CT, and the plug closure was effective and safe. As a result, the patient had a favorable clinical course. On the other hand, the possibility of underexpansion remained and performing additional balloon valvuloplasty was considered a viable option.

## Conclusions

Percutaneous closure is an effective treatment option for controlling PVL and resolving hemolysis in patients implanted with S3UR. The mechanisms and pathophysiology of PVL progression and hemolysis require further investigation.

## Funding Support and Author Disclosures

This report did not receive any grant from any funding agency in the public, commercial, or not-for-profit sectors. Dr Morino has received educational grants from Edwards Lifesciences and lecture fees from Medtronic and Edwards Lifesciences. Dr Ninomiya has received lecture fees from Edwards Lifesciences. Dr Fusazaki has received lecture fees from Medtronic and is a proctor for Medtronic. Dr Kanehama has received lecture fees from Edwards Lifesciences. All other authors have reported that they have no relationships relevant to the contents of this paper to disclose.

## Appendix

Video 1 CT imaging of the aortic valve complex.

Video 2 Final angiography after TAVR. The grade of aortic regurgitation was mild.

Video 3 Transesophageal echocardiography after TAVR. Paravalvular leak was mild.

Video 4 Transesophageal echocardiography one month after TAVR. Paravalvular leak progressed to moderate.

Video 5 CT imaging of the transcatheter heart valve.

Video 6 Transesophageal echocardiography after implantation of vascular plug. Paravalvular leak improved to mild.

## References

[1] Nazif T.M., Cahill T.J., Daniels D. (2021). Real-world experience with the SAPIEN 3 Ultra transcatheter heart valve: a propensity-matched analysis from the United States. Circ Cardiovasc Interv.

[2] Otto C.M., Nishimura R.A., Bonow R.O. (2021). 2020 ACC/AHA guideline for the management of patients with valvular heart disease: a report of the American College of Cardiology/American Heart Association Joint Committee on Clinical Practice Guidelines. J Am Coll Cardiol.

[3] Landes U., Hochstadt A., Manevich L. (2023). Treatment of late paravalvular regurgitation after transcatheter aortic valve implantation: prognostic implications. Eur Heart J.

